# The Levonorgestrel Intrauterine System: Reasons to Expand Access to the Public Sector of Africa

**DOI:** 10.9745/GHSP-D-15-00178

**Published:** 2015-10-28

**Authors:** David Hubacher

**Affiliations:** ^a^​ FHI 360, Durham, NC, USA

## Abstract

The levonorgestrel intrauterine system has: (1) excellent effectiveness, (2) high satisfaction levels, (3) non-contraceptive benefits, and (4) potential to help reinvigorate interest in intrauterine contraception. The time is ripe for ministries and donor agencies to work together to make the product widely available across Africa.

See related article by Jacobstein.

The contraceptive known as the levonorgestrel intrauterine system (LNG IUS) was developed in the 1970s. The product releases small amounts of progestin into the uterus and improves contraceptive action compared with earlier inert plastic devices. The first commercialized LNG IUS set a high standard for future contraceptive development. Relative to other reversible contraceptives, the LNG IUS has the highest effectiveness levels (combining the highest product continuation rates with over 99% efficacy).^1^ The duration of action is 5 years (likely more), and the product provides important non-contraceptive benefits related to how it thins the lining of the uterus and reduces menstrual blood loss; from these effects, it may alleviate and/or prevent iron deficiency anemia. However, because of historically high product cost and a variety of other reasons, the LNG IUS is not widely available to women in resource-poor settings.

The legacy product was first approved in Finland in 1990, in the rest of Europe in the 1990s, and in the United States in 2000^2^; today it is registered in more than 100 countries, and 2014 revenue topped US$900 million (personal communication with Klaus Brill, Bayer HealthCare Pharmaceutical, June 30, 2015, citing the Bayer Annual Report 2014). Because of the product’s great success, even at a high retail price, other companies have recently developed similar technologies in the hopes of entering global markets with lower-cost products. Currently, 5 different pharmaceutical companies make an LNG IUS. Two Indian companies had products approved in India in 2011 and 2012,^3^^,^^4^ and a third Indian company may soon have a product available.^5^ A Belgian company makes an LNG IUS that was approved in Europe in 2014 and in the United States in 2015.^6^

As of 2015, no major international donor agency has issued and executed a tender for purchasing the LNG IUS. Thus, the per unit cost for the public sector is unknown. For drugs to be purchased in large quantities by major international donor agencies, such as the US Agency for International Development (USAID) and the United Nations Population Fund (UNFPA), the products must first be approved by a stringent regulatory authority (e.g., an American, European, or Japanese authority) or by the World Health Organization (WHO) through its Prequalification Programme. Then, the products must be registered at the national level in recipient countries. Currently, only the legacy product meets these criteria for procurement; hopefully, one or more of the other products will soon be eligible as well. Together, these actions may foster eventual purchase of a low-cost product by an international donor agency.

In this paper, I provide a summary of the obstacles to interest in the LNG IUS. Then, I outline 6 main reasons why donor agencies should purchase the LNG IUS and why family planning programs should incorporate the method into their services.

## OBSTACLES TO INTEREST IN THE LNG IUS

Aside from some temporary regulatory obstacles, international donor agencies and foundations have uncertainty over the “value added” with an LNG IUS. The uncertainty involves 3 interrelated issues: (1) high product cost, (2) belief that the current variety of other contraceptive commodities is sufficient, and (3) doubt that an LNG IUS can overcome program barriers to uptake that currently affect the copper intrauterine device (IUD). Each of these real or perceived (current or past) beliefs dampens enthusiasm for taking procurement and program action.

High product cost is a relative concept and a major barrier to LNG IUS procurement. The comparison with the copper IUD, which costs donors less than $1 per unit,^7^ severely limits any notions of purchase. It is hard to imagine that any LNG IUS will reach that price point in the foreseeable future. But the dogged view that the LNG IUS and copper IUD are interchangeable (for the reason that both products are placed in the uterus) is too simplistic; the products’ attributes are vastly different and will attract different users for different reasons. Some compare the LNG IUS to the subdermal implant. Implants currently cost donors $8–$9 per unit; this low price was established in 2013.^8^ However, in the 5-year period preceding this price reduction, the 2 largest donor agencies justified buying more than 8 million implants for approximately $20 each. Thus, while precedence exists to pay higher prices for new technologies, too many other factors prevent the LNG IUS from being procured.

While both the LNG IUS and the copper IUD are types of intrauterine contraception, they have vastly different characteristics and will thus attract different users.

Duration of use also plays into the cost calculations and comparisons. For example, the copper IUD lasts up to 12 years; this fact inappropriately drives perceptions that any new form of intrauterine contraception (i.e., the LNG IUS) needs to match that duration. Of note, the 3-year subdermal implant was not held to the standard of the 5-year implant, yet it has had substantial success and support. Sufficient interest in the LNG IUS may never materialize if donor agencies continue to compare it to the copper IUD or the subdermal implant.

Five primary contraceptive commodities currently make up the backbone of international donor support: injectables, pills, condoms, copper IUDs, and subdermal implants. From a donor perspective, this array enables couples to choose a contraceptive delivery system to suit a variety of personal preferences and needs. The subdermal implant is currently in high supply, due to the volume guarantee arrangement associated with the 2013 price reduction. Programs need to expand access to the product for the arrangement to be successful. If donors and foundations continue to view the 5 main contraceptives as sufficient, then the LNG IUS will never reach its full potential.

Finally, negative perceptions of the copper IUD and the struggles to establish even moderate levels of use in most African countries naturally dampen interest in another intrauterine product such as the LNG IUS. Many factors contribute to the poor uptake of the copper IUD. Previous failed efforts to stimulate uptake of the copper IUD should only be informative of the challenges for a new product, not predictive of a similar destiny.

Previous challenges with stimulating uptake of copper IUDs should inform new product rollout but should not be predictive of a similar destiny.

## SIX REASONS TO INVEST IN THE LNG IUS

The LNG IUS is a unique type of contraceptive that combines 2 key features: intrauterine placement and use of a proven and safe progestin. It will attract new users and stimulate programs. For the reasons outlined below, the LNG IUS should be procured by the international donor community.

### 1. Recently Placed on WHO’s Essential Medicines List

WHO recently placed the LNG IUS on the Essential Medicines List.^9^ Thus, in the eyes of WHO, the LNG IUS is now considered one of the most “efficacious, safe, and cost-effective medicines for priority conditions.”^9^ Priority conditions are “selected on the basis of current and estimated future public health relevance, and potential for safe and cost-effective treatment.” Now, according to WHO, a health care system is not meeting basic needs without the LNG IUS on the formulary.

According to WHO, a health care system is not meeting basic needs without the LNG IUS on the formulary.

### 2. Well Accepted Worldwide

Worldwide, user satisfaction with the LNG IUS is consistently high. A study in 18 different countries in Asia and Europe found that 95% of LNG IUS users were satisfied with the product.^10^ Compared with the subdermal implant, the LNG IUS is often better tolerated. For example, in Australia, 3-year continuation rates of the LNG IUS and the etonogestrel subdermal implant were 73% and 53%, respectively.^11^ In the United States, the LNG IUS continuation rate at 24 months was found to be significantly higher than that of the subdermal implant (79% versus 69%, respectively).^12^ In the only head-to-head comparison of the levonorgestrel subdermal implant and the LNG IUS, the subdermal implant had a removal rate for irregular bleeding of 26.8% at 36 months compared with 3.3% for the LNG IUS.^13^ Prolonged bleeding consistently affected 20% to 40% of implant users at different follow-up times, but the problem did not exceed 10% for LNG IUS users.

Research in Africa also shows good prospects for the LNG IUS. In Kenya, 16% of family planning clients chose the LNG IUS when given the opportunity; only 3% wanted the copper IUD.^14^ Kenyan providers cited non-contraceptive benefits (reduction of menstrual blood loss, alleviation of anemia) as important reasons for recommending the LNG IUS to clients.^15^ In a prospective cohort study, approximately 90% of women in Kenya were still using the LNG IUS after 12 months.^16^ A survey in South Africa found that 75% of respondents were positive toward a product such as the LNG IUS if it would reduce menstrual bleeding.^17^ In Ghana, LNG IUS users and providers had similar high opinions of the product.^18^ In Ghana and 8 other countries in sub-Saharan Africa, the International Contraceptive Access (ICA) Foundation has donated thousands of LNG IUS products since 2003.^19^^,^^20^

### 3. Unique/Advantageous Delivery System and Important Non-Contraceptive Benefits

No other hormonal method releases progestin directly into the uterus; this results in a predominately local effect with highly efficacious triple-action mechanisms on the cervix, endometrium, and ovaries to prevent pregnancy. Moreover, the level of levonorgestrel released from the LNG IUS is steady and slow, which minimizes side effects. In contrast, short-acting hormonal methods have abrupt dosing effects and higher levels of circulating hormones that create peaks and troughs of systemic levels; for many users, this causes intolerable side effects, leading to high discontinuation rates.

The slow and steady release of hormone directly into the uterus with the LNG IUS results in high efficacy and minimal side effects.

Non-contraceptive benefits also make the LNG IUS unique; the benefits are linked to how the LNG IUS shrinks the endometrium and minimizes uterine bleeding. The LNG IUS is a proven treatment for heavy menstrual blood loss and is more effective than all other non-hysterectomy approaches.^21^ The LNG IUS also decreases bleeding among women with uterine fibroids.^22^ Finally, the LNG IUS has very promising prevention actions against a number of conditions, including endometriosis, uterine fibroids, endometrial hyperplasia, endometrial cancer, and peri-menopausal menstrual disturbances.^23^

The LNG IUS is a proven treatment for heavy menstrual blood loss.

The LNG IUS may help anemic women; this is probably the most important potential non-contraceptive benefit for women in resource-poor countries. Through the mechanism of reducing menstrual blood loss, the LNG IUS can help anemic women retain up to 19 grams of iron each month.^24^ This amount, when accumulated over many months, might increase iron stores and prepare women for healthy pregnancy when the time comes. Anemia affects approximately 46% of women of reproductive age in sub-Saharan Africa and Southeast Asia,^25^ and it contributes to 20% of all maternal deaths.^26^ Prolonged anemia after pregnancy, short birth intervals, and even poor nutritional status prior to a first pregnancy increase the risks of poor health outcomes.

By reducing menstrual blood loss, the LNG IUS might increase women’s iron stores.

Because of these unique attributes, the LNG IUS is not interchangeable with the copper IUD. In Kenya, nearly a third of LNG IUS acceptors said they would have chosen a short-acting method if the LNG IUS were not available, and only 21% would have chosen the copper IUD.^14^ In the United States, approximately 80% of intrauterine contraceptive use is attributable to the LNG IUS and 20% to the copper IUD; the CHOICE study demonstrated that women overwhelmingly wanted the LNG IUS in a 4 to 1 ratio over the current copper IUD product.^27^

### 4. Opportunity to Activate Some Countries

The best example of rapid uptake of the LNG IUS is from the United States. In this mature contraceptive market, the LNG IUS quickly reinvigorated interest in intrauterine contraception.^28^ Between 1995 and 2013, the proportion of all contraceptive users using intrauterine contraception in the United States rose from less than 1% to over 10%.^29^ Today intrauterine contraception is more popular in the United States than any new delivery system introduced since 1992, including the injectable DMPA (1992), the vaginal ring (2001), the patch (2002), and the etonogestrel subdermal implant (2006).^30^^,^^31^ The general rise in intrauterine contraceptive use was also seen in federally funded family planning programs, in which provision increased from 48,000 intrauterine contraceptive services in 1999 to more than 270,000 in 2011.^32^

Ministries of Health recognize the importance of expanding access to long-acting reversible contraception, particularly since sterilization services are more difficult to provide. In the 1980s, many countries put the newly available copper IUD at the center of their family planning programs. Egypt, Mexico, Turkey, and Vietnam experienced tremendous fertility transition in one generation by making the IUD widely accessible. Today, Ethiopia’s Ministry of Health is using health extension workers to make subdermal implants widely available; this may result in similar patterns of uptake that were observed in countries where the copper IUD became popular. Other countries will focus on the LNG IUS when it becomes available. No one can predict which countries will launch the LNG IUS and make it successful, just as nobody predicted which countries would succeed with copper IUDs and implants.

The international family planning community is ready to support the LNG IUS. At the biennial International Conference on Family Planning in Ethiopia in 2013, a small survey of 30 conference participants found that 100% of respondents with knowledge of the LNG IUS supported the idea of making the product available in public-sector clinics worldwide (personal communication with Heather Vahdat, FHI 360, November 11, 2014). When asked why they would support the introduction of an LNG IUS, responses fell primarily into 2 categories: benefits offered by the method (e.g., long-acting, fewer side effects, reduced bleeding) and to support expansion of the method mix/choice for women.

### 5. Many Women in sub-Saharan Africa Want to Use Intrauterine Contraception

NGOs consistently show high uptake of IUDs in the same public-sector clientele where ministries fail. The difference is that NGO providers have the knowledge, skills, job expectations, and proper support to make IUD services available. For example, between 2008 and 2012 Marie Stopes International more than tripled the number of IUDs it provided in sub-Saharan Africa, from 69,087 insertions to more than 215,000.^33^^,^^34^ The interest in long-acting methods overall is growing.^35^

Introduction of the LNG IUS is a new opportunity for Ministries of Health and providers in the public sector to establish successful, demand-driven intrauterine contraception services. As demonstrated in the United States, a single intrauterine product with a proven track record and high acceptability can stimulate provision of services, even in the public sector. With a new product such as the LNG IUS, public-sector providers can develop the skills and confidence to insert IUDs and serve the clientele who currently have latent demand for services. Thus, the LNG IUS may be the easiest route to establish consistent access to the copper IUD.

### 6. Need for More Highly Effective Options

WHO lists IUDs (copper IUDs and the LNG IUS) and subdermal implants as the most effective reversible forms of contraception.^36^ Only subdermal implants and intrauterine contraception can compensate for lack of access to sterilization services. For spacing births, implants and intrauterine contraception eliminate the risks of unintended short birth intervals. In public-sector facilities throughout sub-Saharan Africa, the current array of contraceptive choices is imbalanced in favor of the least effective, short-acting options. Thus, women who need permanent or long-term protection face difficult challenges over many years. For many countries, the copper IUD has not contributed much toward reducing national levels of unintended pregnancy and fertility. New products such as the LNG IUS are needed to attract new users to highly effective, long-acting contraception.

Intrauterine contraception and implants are the most effective reversible forms of contraception.

Adolescents also need more effective options like the LNG IUS. Preventing unintended pregnancy in this population (especially first pregnancies) can help young women achieve other goals they may have (e.g., education, employment). Consensus is building that long-acting reversible contraceptives should be easily available to sexually active young women.^37^^,^^38^

### Summary

The LNG IUS is not just another, interchangeable contraceptive. This fact should help justify taking procurement and program action for the public sector in sub-Saharan Africa. For many decades, the obstacles to procurement appeared insurmountable. Today, however, international donors have better information and more opportunities to make large-scale procurement a reality. With renewed emphasis and funding for family planning, the timing couldn’t be better. Women in resource-poor countries will benefit tremendously from the LNG IUS; worldwide equity in access to the technology is needed.

## Notes

**Peer Reviewed**
